# Feasibility of Contrast-Enhanced Spectral Mammography in Japan: A Pilot Study

**DOI:** 10.31662/jmaj.2026-0014

**Published:** 2026-05-08

**Authors:** Yuko Ito, Tomoyuki Fujioka, Kazunori Kubota, Yuka Yashima, Anna Tabei, Haruka Tanaka, Toshihisa Ogawa, Tsuyoshi Nakagawa

**Affiliations:** 1 Department of Radiology, Dokkyo Medical University Saitama Medical Center, Koshigaya, Japan; 2 Department of Breast and Endocrine Surgery, Dokkyo Medical University Saitama Medical Center, Koshigaya, Japan; 3 Department of Breast Surgery, Dokkyo Medical University, Mibu, Japan

**Keywords:** contrast-enhanced spectral mammography (CESM), magnetic resonance imaging (MRI), breast cancer, mammography, dense breast, contrast agent

## Abstract

**Introduction:**

Contrast-enhanced spectral mammography (CESM) provides diagnostic capability comparable to dynamic contrast-enhanced magnetic resonance imaging (DCE-MRI); however, in Japan, CESM is not routinely performed because the use of iodinated contrast agents for CESM is not approved. Evidence on CESM from Japan is therefore scarce. This study assessed the feasibility, diagnostic performance, and patient experience of CESM in Japanese patients with breast cancer.

**Methods:**

This study enrolled women aged 20-80 years with histologically confirmed breast cancer who were scheduled for surgery. CESM and DCE-MRI were performed within two months preoperatively. Images were independently interpreted by a breast radiologist. Tumor size, Breast Imaging Report and Data System (BI-RADS) category, and breast density were assessed and compared with postoperative pathology. Average glandular dose (AGD) and contrast-related reactions were evaluated. Patients answered questions regarding examination time, comfort, positioning, and preferred modality. Pearson correlation coefficients were calculated to assess the relationship among tumor sizes measured on CESM, DCE-MRI, and pathology.

**Results:**

A total of nine patients (11 malignant lesions) were analyzed. CESM detected 10 lesions (detection rate 90.9%) and DCE-MRI detected all lesions (100%). Tumor size measured by CESM (r = 0.95) and DCE-MRI (r = 0.94) showed strong correlation with pathology. AGD averaged 3.1 mGy, and no allergic reactions occurred. Patients preferred CESM primarily due to shorter examination time, while noise and long duration were the main disadvantages of DCE-MRI.

**Conclusions:**

CESM demonstrated high diagnostic performance that was comparable to DCE-MRI and superior to mammography, particularly for dense breasts. Considering its shorter examination time and favorable patient acceptance, CESM may serve as a valuable diagnostic option in Japan, thus warranting further clinical research.

## Introduction

Contrast-enhanced spectral mammography (CESM) is a diagnostic imaging tool to evaluate breast cancer based on a dual-energy system for mammography acquisition, after intravenous administration of iodinated contrast agents. It is well known that CESM has a higher diagnostic performance than mammography (MMG) ^(1)^. CESM was first approved by the U.S. Food and Drug Administration (FDA) in 2011. In Japan, the regulatory approval for iodinated contrast agents for CESM was granted in 2011; however, the procedure is still not covered by insurance as of November 2025.

Dynamic contrast-enhanced magnetic resonance imaging (DCE-MRI) is now strongly recommended to evaluate breast cancer ^(2), (3)^. However, DCE-MRI has several limitations. It is expensive and time-consuming; patients with metallic hardware cannot undergo MRI and dialysis patients are unable to use MRI contrast agents.

CESM has been reported to be equally as capable as DCE-MRI in assessing the detection of breast malignancies in patients with breast cancer, which is necessary for preoperative staging and treatment planning ^(4), (5), (6)^. There have also been reports comparing the patient experience of CESM and DCE-MRI ^(7), (8)^. When the diagnostic performance of examination is equivalent, trial examination with high patient satisfaction will play an important role in ensuring the quality of medical care.

There are only limited reports on CESM in Japan, and to the best of our knowledge, neither there have been reports comparing the diagnostic performance of CESM with that of DCE-MRI nor there have been any reports evaluating patient experience measures in this context. The purpose of this pilot study is to consider the benefits and harms of CESM if it becomes an established examination for patients with breast cancer in Japan. We evaluated the diagnostic performance of CESM and DCE-MRI in comparison with pathological findings. We also assessed breast composition, average glandular dose (AGD), allergic reactions caused by contrast agents, and answers to some questions about CESM and DCE-MRI regarding which examination would be better for patients with breast cancer.

## Materials and Methods

This study was approved by the Institutional Review Board (IRB) of Dokkyo Medical University (approval date: March 13, 2017; the 9th IRB meeting; approval number: 1664) and was conducted between April 2017 and December 2018. In this period, 1,380 and 338 patients underwent MMG and DCE-MRI, respectively.

CESM is not covered by the Japanese National Health Insurance system, either at the time of the study or at present, and is not performed as part of routine clinical practice in Japan. In this study, CESM examinations were conducted exclusively under an IRB-approved research protocol.

To participate, patients had to be 20 to 80 years old, who have been diagnosed with breast cancer histologically and who planned to undergo surgery for breast cancer. The exclusion criteria are as follows: surgical history of index imaging range; implanted pacemakers or defibrillator and ventriculoperitoneal shunt; pregnancy or possible pregnancy and lactation; allergy to contrast agent; history of neo-adjuvant therapy; an estimated glomerular filtration rate less than 45 mL/min/1.73 m^3^; and proven positive margin of resected specimen in the surgery. All the patients underwent CESM and DCE-MRI within two months before surgery.

### CESM protocol

CESM examinations were performed with a digital mammography device (Senographe Essential; GE HealthCare, Chicago, IL, USA), allowing dual-energy CESM acquisitions. Iopamidol (Oypalomin; Fuji Pharma, Tokyo, Japan) was used as the CESM contrast agent. The contrast medium at 1.5 mL/kg body weight (BW) and at a rate of 3 mL/sec was administered using a power injector. Two minutes after injection, craniocaudal (CC) and mediolateral oblique (MLO) views of the abnormal breast were acquired within five minutes. Next, CC and MLO views of the normal breast were acquired within five minutes, and each patient’s CESM images were ready within 10 minutes. Both low-energy and weighted subtraction images in each view were transferred to the workstation for radiologist review.

### DCE-MRI protocol

The DCE-MRI examinations were carried out on SIEMENS MAGNETOM Skyra 3T (Simens; Bayern, Germany). All the patients were in a supine position with no breast compression, using breast-dedicated coils. Gadobutrol (Gadovist; Bayer, Leverkusen, Germany) was used as the contrast medium and was injected at a rate of 0.1 mL/sec with a dose of 0.1 mmol/kg BW. The breast MRI protocol included T1-weighted and T2-weighted pre-contrast fat-suppressed images in the transverse plane. Dynamic imaging including one pre-contrast and four cycles of post-contrast imaging, diffusion-weighted imaging with apparent diffusion coefficient, and reconstructed sagittal post-contrast images also obtained. After completion of the acquisitions, all images are automatically sent to the workstation for radiologist review.

### Image analysis

The images obtained with the two imaging techniques (CESM and DCE-MRI) were evaluated independently, with each modality reviewed separately by a radiologist with 10 years of experience in breast imaging, without access to the results of the other modality. The radiologist assessed index lesions, breast parenchymal enhancement (BPE), and breast composition on MRI according to the 5th edition of the American College of Radiology Breast Imaging Report and Data System (ACR BI-RADS). Strong enhancement compared with normal breast tissue was considered a positive assessment on CESM. The maximum diameter of the suspected malignant lesions was measured in both examinations. After the evaluation of each imaging investigation, all the findings were compared with postoperative histological results.

The total mean AGD of bilateral breasts was calculated. Allergic reactions were checked for both examinations within one week after the procedure by reviewing the patients’ electronic medical records.

After all the patients had completed both examinations, they answered three questions about the examinations. They were also free to describe what they liked and disliked about the two examinations, and answered a final question about which examination they would prefer if they were having a breast examination.

The detection rates of CESM and DCE-MRI were calculated with two-sided 95% confidence intervals (CIs), using the Clopper-Pearson exact method. The measured data were presented as a mean ± standard deviation (SD). Analysis using Pearson correlation coefficient (PCC) was based on the maximum tumor diameter measured on CESM images, DCE-MRI images, and pathological findings. A p-value of <0.01 was considered statistically significant. Analyses were performed with IBM SPSS Statistics version 27 (IBM Corp.; Armonk, NY, USA).

## Results

A total of 11 women provided consent for the study. Two patients were excluded from the image-based evaluation for the following reasons: one underwent CESM and the images could not evaluate the suspicious lesions because of motion artifact, and one had a proven positive margin of resected specimen in the surgery. Nine patients remained, with a mean age of 59 years (range, 36-78 years).

Table 1 shows the imaging and pathological characteristics of the patients. Histopathologically, there were 11 malignant lesions. In seven patients, single breast lesions were detected, while in two patients two malignant lesions were diagnosed. Of the 11 lesions, 10 (91%) were invasive ductal carcinoma and 1 (9%) was noninvasive ductal carcinoma. Compared with histopathological findings, one index tumor was undetected on CESM and all the index tumors were detected on DCE-MRI. Detection rates for CESM and DCE-MRI were 91% (10/11; 95% CI, 58.7%-99.8%) and 100% (11/11; 95% CI, 71.5%-100%), respectively. The mean lesion size was 15.5 mm ± 8.3 (SD) on CESM and 16.4 mm ± 9.3 (SD) on DCE-MRI. The smallest lesion measured on CESM was 7.1 mm, compared to 7.3 mm on DCE-MRI (Table 2).

**Table 1.** Imaging and Pathological Characteristics of the Patients.

		DCE-MRI				CESM			Pathology						
Patient No	Case No	BPE	Pattern	Size (mm)	Category	Breast density	Pattern	Size (mm)	Histological type	Size (mm)	ER	PgR	HER2	NG	Ki-67 (%)
1	1	minimal	mass	27.0	5	C	mass	22.6	IDC	19	+	+	+	2	20
2	2	minimal	mass	8.2	5	B	mass	11.7	IDC	9	+	－	－	3	37
	3		mass	7.3	4c		mass	7.3	IDC	5	+	－	－	3	N. A
3	4	mild	mass	22.0	5	C	mass	18.6	IDC	20	－	－	+	3	60
4	5	minimal	mass	32.0	5	B	mass	30.0	IDC	35	+	+	－	3	30
5	6	minimal	mass	26.0	5	C	mass	26.1	IDC	22	+	+	－	2	30
	7		NME	3.0	3		undetected	undetected	DCIS	7	N. A	N. A	N. A	N. A	N. A
6	8	minimal	mass	12.3	5	B	mass	12.3	IDC	12	+	－	－	1	1
7	9	minimal	mass	10.8	5	B	mass	7.9	IDC	7	+	+	－	1	N. A
8	10	minimal	mass	10.4	5	C	mass	11.3	IDC	13	+	+	+	1	15
9	11	mild	mass	8.1	5	C	mass	7.1	IDC	8	+	+	－	1	5

BPE: background parenchymal enhancement; CESM: contrast-enhanced spectral mammography; DCE-MRI: dynamic contrast-enhanced magnetic resonance imaging; DCIS: ductal carcinoma in situ; ER: estrogen receptor; HER2: human epidermal growth factor receptor 2; IDC: invasive ductal carcinoma; N.A.: not applicable; NG: nuclear grade; NME: non-mass enhancement; PgR: progesterone receptor. Breast density B: Scattered; Breast density C: Heterogeneously dense.

**Table 2.** Detection Rate and Size Estimation between CESM and DCE-MRI.

	CESM	DCE-MRI
Detection rate	90.9% (10/11; 95% CI, 58.7-99.8%)	100% (11/11; 95% CI, 71.5-100%)
The mean size of index lesions	15.5 mm ± 8.2 (SD)	16.4 mm ± 9.3 (SD)
The smallest size of index lesions	7.1 mm	7.3 mm

CESM: contrast-enhanced spectral mammography; CI: confidence intervals; DCE-MRI: dynamic contrast-enhanced magnetic resonance imaging; SD: standard deviation.

Evaluation of tumor size using the PCC showed strong correlations between CESM and pathological findings (r = 0.95, p < 0.01), between DCE-MRI and pathological findings (r = 0.94, p < 0.01), and between CESM and DCE-MRI (r = 0.97, p < 0.01) (Table 3).

**Table 3.** Pearson’s Correlation Coefficient Using the Maximum Diameter of the Tumor.

	r	p
CESM vs Pathology	0.95	<0.01
DCE-MRI vs Pathology	0.94	<0.01
CESM vs DCE-MRI	0.97	<0.01

CESM: contrast-enhanced spectral mammography; DCE-MRI: dynamic contrast-enhanced magnetic resonance imaging.

On DCE-MRI, 9 lesions were classified as BI-RADS category 5, 1 lesion as category 4c, and 1 lesion as category 3. BPE was minimal for seven out of nine patients and mild for two out of nine patients. On CESM, breast density was scattered for four out of nine patients and heterogeneously dense for five out of nine patients (Table 1). AGD was 3.1 mGy ± 0.95 (SD) on the right breast and 3.1 mGy ± 0.95 (SD) on the left breast. No apparent post-examination allergic reactions were observed for either examination.

**Figure 1.**
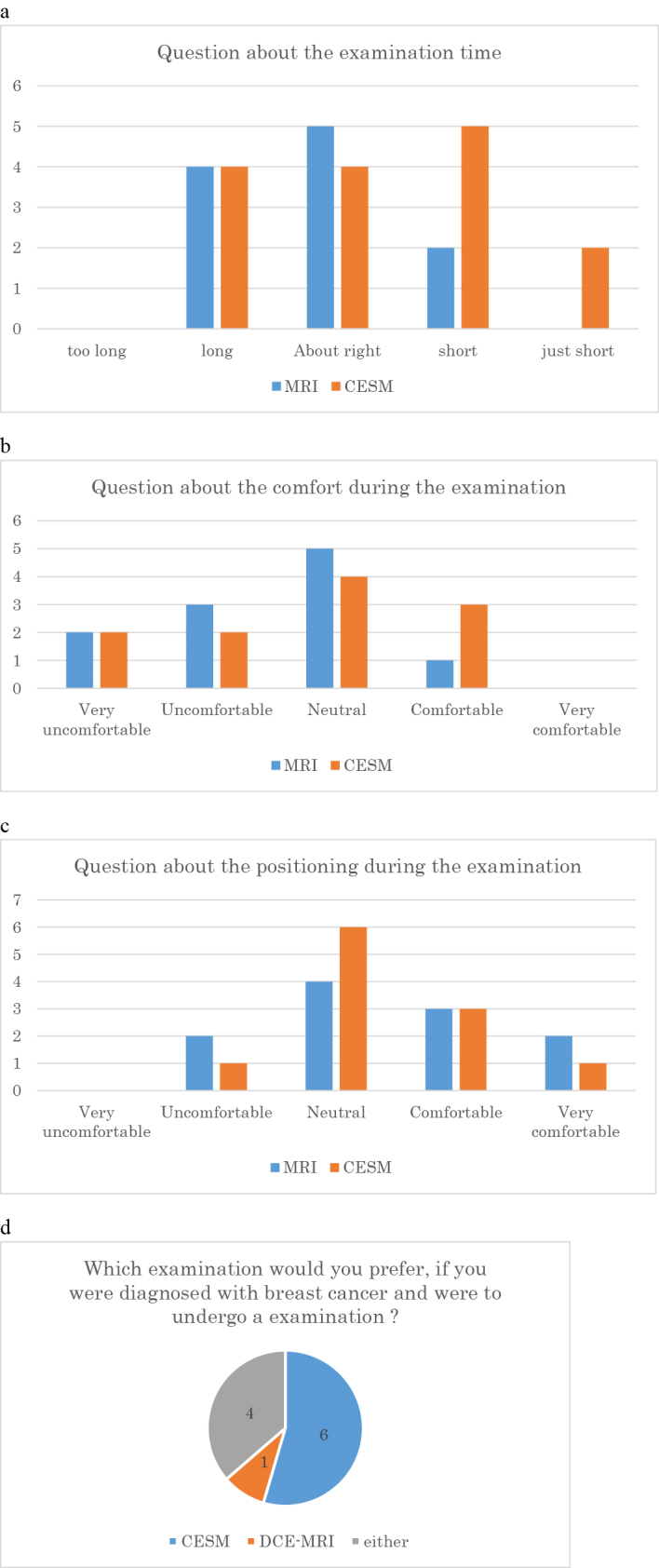
Summary of patient responses. Figure 1a shows the responses regarding examination time, Figure 1b shows the responses regarding comfort during the examination, Figure 1c shows the responses regarding positioning comfort, and Figure 1d shows the patients’ preferred modality if they were to undergo a breast examination.

Figure 1 presents the patients’ answers regarding CESM and DCE-MRI, including questions about examination time (Figure 1a), comfort during the examination (Figure 1b), positioning comfort (Figure 1c), and their preferred modality if they were to undergo a breast examination (Figure 1d). Table 4 summarizes the open-ended comments describing favorable and unfavorable aspects of both CESM and DCE-MRI.

**Table 4.** Open-Ended Comments on CESM and DCE-MRI.

Modality	Aspect	Number of applicable / all
CESM	favorable	Short examination time (5/11)
		Comfortable body position during the examination (1/11)
	unfavorable	Concerns about CESM because of unpleasant experience with mammography (1/11)
		Discomfort during injection (1/11)
		Pain during breast compression (2/11)
DCE-MRI	favorable	Comfortable body position during the examination (1/11)
		Less discomfort during injection compared to CESM (1/11)
	unfavorable	Long examination time (2/11)
		Noise during the examination (3/11)
		Discomfort due to body position during the examination (1/11)

CESM: contrast-enhanced spectral mammography; DCE-MRI: dynamic contrast-enhanced magnetic resonance imaging.

## Discussion

This study is a pilot investigation evaluating the feasibility of CESM in Japan. In Japan, the use of iodinated contrast agents for CESM is not approved, and CESM itself is not performed in routine clinical practice. Consequently, reports on CESM from Japan are extremely limited, and available data remain scarce.

Previous domestic studies have been limited in scope. Mori et al. ^(9)^ compared CESM with conventional full-field digital MMG in women with dense breasts and demonstrated improved diagnostic accuracy of CESM over standard MMG in this population. Okada et al. ^(10)^ investigated the feasibility of performing CESM immediately after contrast-enhanced computed tomography (CT), without additional contrast administration, suggesting a potential workflow advantage in selected clinical settings.

However, to the best of our knowledge, neither there have been reports in Japan directly comparing the diagnostic performance of CESM with DCE-MRI nor have there been any studies evaluating patient experience measures associated with these examinations. In this context, our study is the first in Japan to assess both the diagnostic performance of CESM in comparison with that of DCE-MRI and patient-reported experience measures, representing a significant strength and providing valuable insights into the potential clinical utility and safety of CESM in the Japanese healthcare setting.

In our study, tumor size measured by both CESM and DCE-MRI were strongly correlated to pathology. The detection rates of CESM and DCE-MRI for the detection of breast carcinoma was 91% and 100%, respectively. Previous studies have reported a sensitivity for detecting index lesion of 94%-100% and 93%-95% for CESM and DCE-MRI, respectively. They have also reported that specificity is higher for CESM than that for DCE-MRI ^(1), (4), (6)^. CESM has also been reported to be as reliable as DCE-MRI in assessing tumor size before treatment and after neoadjuvant chemotherapy ^(4), (5), (11), (12)^. In addition, Patel et al. ^(13)^ and Cheung et al. ^(14)^ suggested that CESM is superior to DCE-MRI in terms of medical economy. Patel et al. ^(13)^ reported that CESM is performed at about 25 % of the cost of DCE-MRI. The use of CESM as an alternative to DCE-MRI is likely to be beneficial in terms of healthcare costs, especially for patients who cannot afford a DCE-MRI.

**Figure 2.**
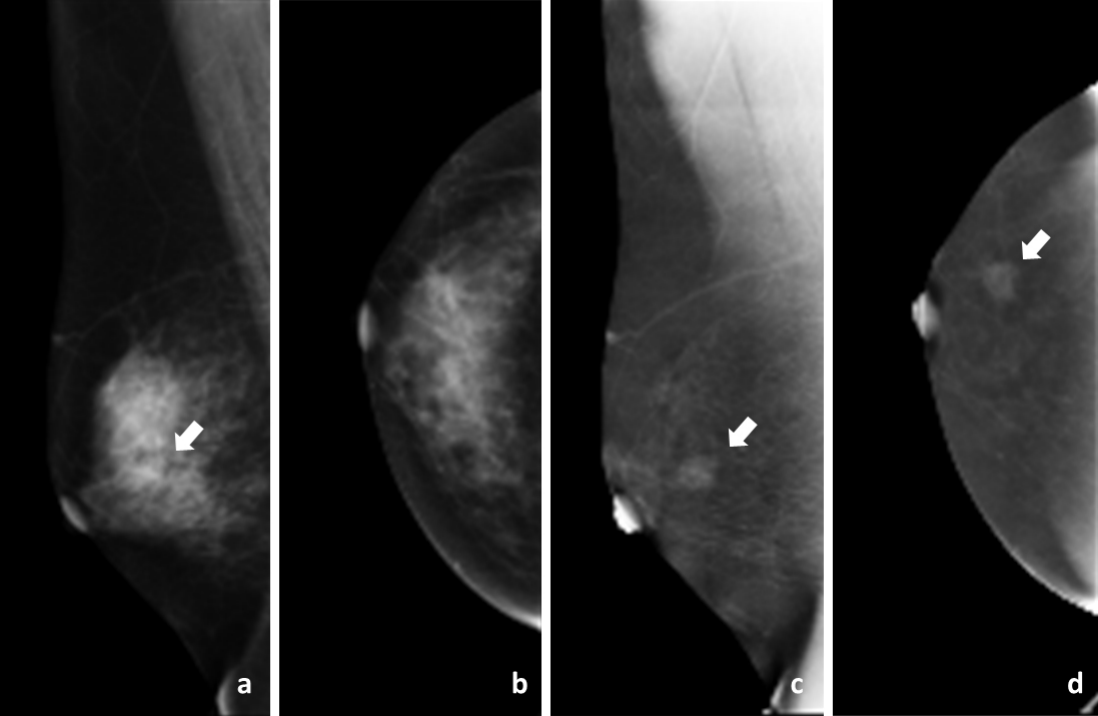
A case in which CESM was useful for detecting breast cancer. Figure 2a shows a focal asymmetric density in the middle region on the mediolateral oblique view (arrowhead) of mammography in a heterogeneously dense breast, while Figure 2b shows no notable abnormality on the corresponding craniocaudal view. Figure 2c shows an enhancing 11.3-mm lesion involving the outer and middle regions on the mediolateral oblique view (arrowhead) of CESM, and Figure 2d shows the same lesion on the craniocaudal view (arrowhead). Subsequent surgery was performed, and pathological examination revealed a 13.0-mm invasive ductal carcinoma corresponding to the outer lesion. CESM: contrast-enhanced spectral mammography.

CESM showed a better lesion detection and size estimation than MMG, in comparison to postoperative histology, and may be useful in detecting the lesions in dense breasts on MMG ^(6), (15), (16), (17)^. In our study, there was one case where a malignant tumor that was not apparent on MMG alone could be diagnosed by the addition of CESM and DCE-MRI. It was an invasive ductal carcinoma, and the breast was classified as heterogeneously dense on MMG. The lesion was evaluated as focal asymmetric density on MMG, and was described as a 10 mm contrasted area on CESM and DCE-MRI (Figure 2). Kim et al. ^(18)^ reported that compared with MMG alone, there was no change in sensitivity between 10 readers when CESM was added to MMG, but specificity increased in seven out of 10 subjects, including two with significant differences. Higher specificity has the benefit of reducing the unnecessary invasive procedures for the patient.

It is known that a high percentage of Japanese women have dense breasts ^(19), (20)^, and CESM is superior to MMG in diagnosing breast cancer in Japanese women with dense breasts ^(9)^. The sensitivity of CESM and MMG was reported as 86.2% and 94.1%, respectively, and the specificity of CESM and MMG was reported as 53.4% and 85.9%, respectively ^(9)^. The incidence of breast cancer in dense breasts has been reported in the past, and it showed that dense breasts tended to have a higher risk of developing breast cancer than fatty breasts ^(20), (21), (22), (23)^. In this study, only four patients were classified as having scattered fibroglandular density and five patients as having heterogeneously dense breasts, resulting in a relatively small proportion of dense breasts in our study population. Therefore, our data are insufficient to draw firm conclusions regarding the diagnostic performance of CESM in Japanese women with dense breasts. Because the number of cases in this study was limited, it was not feasible to perform detailed analyses based on breast density, MRI BI-RADS findings, or category-specific comparisons between CESM and DCE-MRI. Future research with larger cohorts will be needed to enable such comprehensive evaluations.

In several countries, DCE-MRI is useful in screening patients with a high lifetime incidence of breast cancer ^(24), (25), (26), (27)^. In recent years, there have been reports of the usefulness of CESM for screening such types of patients ^(28), (29)^. There is no established screening system for Japanese women with a high lifetime incidence of breast cancer, who have genetic abnormalities of hereditary breast cancer such as hereditary breast and ovarian cancer syndrome. In the future, breast cancer screening in Japan is expected to shift toward a more personalized, risk-based approach. To support this transition, further research involving diverse patient groups is essential. Incorporating CESM into such investigations may contribute to high-quality care by appropriately balancing benefits and potential risks.

According to the results of open-ended comments in our study, most patients preferred the short examination time in CESM (5 of 11), and most patients disliked the noise during the DCE-MRI (3 of 11). The next most common comments were that the breast compression in CESM and the long examination time in DCE-MRI were undesirable (2 of 11). When asked which examination they would prefer to have if they were diagnosed with breast cancer, 10 of 11 patients answered they would prefer CESM or neither, and only one patient answered they would prefer DCE-MRI. Previous reports have also suggested that patients, even high-risk patients, prefer the experience of CESM to DCE-MRI ^(7), (8)^. Patients would benefit from the option of CESM as a detailed examination for breast cancer.

In terms of disadvantages, the first one is radiation exposure. United States FDA states an upper limit of 3.0 mGy for the radiation dose of each breast in Mammography Quality Standards Act Regulations and this value is the same as the value set by the International Atomic Energy Agency (IAEA). CESM can be performed within the prescribed AGD ^(30)^. On the other hand, considering that MMG for screening purposes is disadvantageous for those under 40 years of age, the conditions under which CESM can be used for screening purposes in young people should be carefully examined ^(31)^.

The next possible harm is the side effects caused by contrast agents. Adverse reactions of using non-ionic contrast agents have been reported in 0.6% of patients who underwent the examination, of which 2% had a severe reaction such as no-response or semi-response ^(32)^. Although informed consent for the use of contrast agents in CESM is necessary, we consider that the side effects of contrast agents are unlikely to cause significant harm and that the risk associated with the use of contrast agents can be further reduced by interviewing patients about their asthma and history of adverse reactions on non-ionic contrast agents.

**Figure 3.**
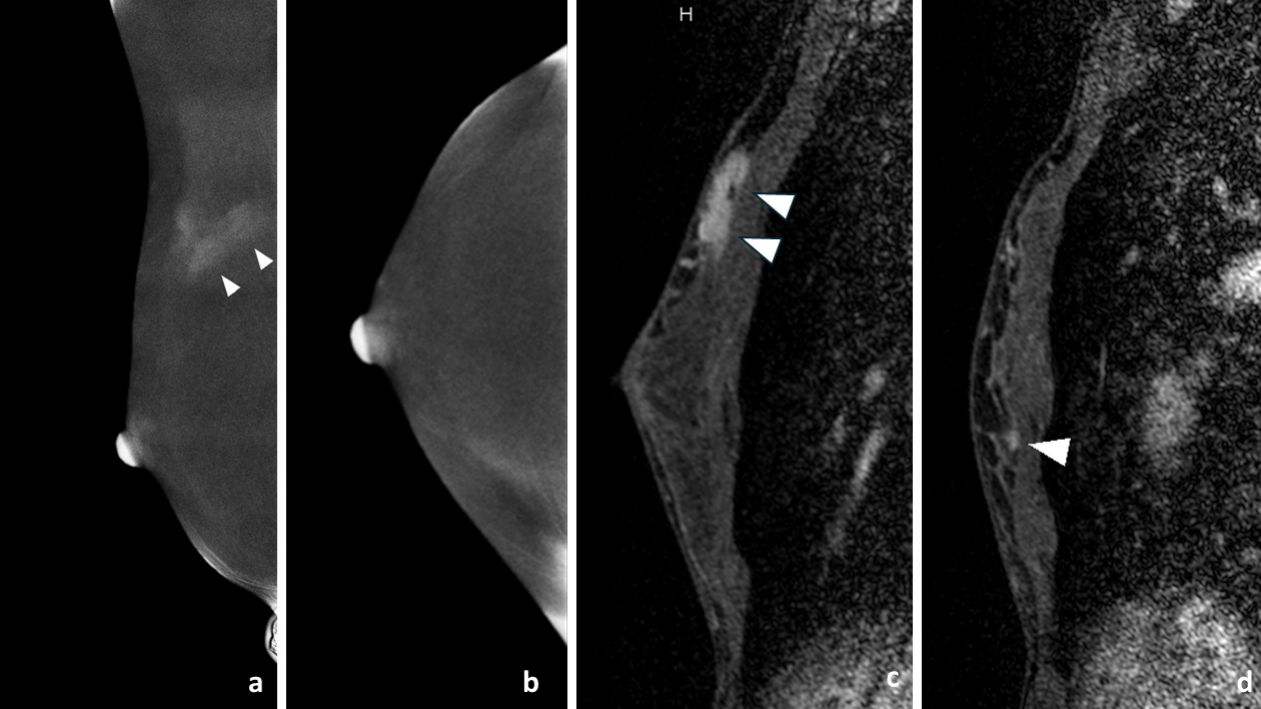
A case in which CESM resulted in a false-negative finding. Figure 3a shows an enhancing lesion measuring 26.0 mm in the upper region on the mediolateral oblique view (arrow). In contrast, Figure 3b shows no enhancing lesion on the craniocaudal view of CESM, because the lesion was outside the imaging field of view. Figure 3c shows an enhancing lesion measuring 26.1 mm in the upper region on the sagittal view (arrow), and Figure 3d shows an enhancing lesion measuring 3 mm in the outer region on the sagittal view (arrow) of dynamic contrast-enhanced magnetic resonance imaging. Subsequent surgery was performed, and pathological examination revealed a 22.0-mm invasive ductal carcinoma corresponding to the upper lesion and a 7-mm noninvasive ductal carcinoma corresponding to the outer lesion. CESM: contrast-enhanced spectral mammography.

In our study, one malignant lesion missed on CESM was a 7-mm noninvasive ductal carcinoma (Figure 3a-d). It was described as a 3-mm area with enhancement on DCE-MRI. Noninvasive ductal carcinoma lacking angiogenesis has been reported to show weak contrast enhancement or non-mass enhancement on DCE-MRI ^(3)^. It is conceivable that a similar mechanism may have occurred in CESM or ionization of contrast agents influences the enhancement so that the difference of contrast agents between CESM and DCE-MRI might influence the appearance of those examinations ^(33)^. Dromain et al. ^(34)^ reported that the same pattern of typical MRI kinetic curve for malignancy was only observed in four out of the 20 malignant lesions of the study, and suggested that the differences between kinetic curves observed using dynamic CESM and DCE-MRI are probably due to breast compression that may alter blood flow even if it is reduced. Reports on CESM are gradually accumulating overseas and the focus of these reports is diverse. It is important to examine the results of these studies and to conduct further research in Japan ^(35)^.

This study had several limitations. First, our sample size was limited to 11 women. Second, only one radiologist evaluated images of both examinations in a single center. Third, the timing of the images was not determined by considering the last menstrual period and it may cause bias in images. Fourth, the questionnaire used in this study was not a validated survey instrument.

### Conclusions

CESM is a better diagnostic examination for evaluating breast cancer than MMG, especially in dense breasts, and is comparable to the diagnostic performance of DCE-MRI. In addition to this, CESM offers advantages over DCE-MRI in terms of cost-effectiveness and patient satisfaction, not only for patients who cannot undergo DCE-MRI. In Japan, the benefits of CESM are expected to outweigh the harms, and CESM may be widely used in Japan for breast cancer order-made screening and examinations.

## Article Information

### Acknowledgments

We used GPT-5.2 (https://chat.openai.com/) for Japanese-English translation and English proofreading. The generated text was reviewed, revised, and approved by the authors.

### Author Contributions

Conceptualization, Yuko Ito and Tomoyuki Fujioka; methodology, Yuko Ito; investigation, Yuka Yashima, Anna Tabei and Haruka Tanaka; resources, Toshihisa Ogawa and Tsuyoshi Nakagawa; and supervision, Kazunori Kubota. All authors have read and agreed to the published version of the manuscript.

### Conflicts of Interest

None

### IRB Approval Code and Name of the Institution

Study approved by the Institutional Review Board of Dokkyo Medical University (approval date: March 13, 2017; the 9th IRB meeting; approval number: 1664).
